# Clinical and experimental study of a terahertz time-domain system for the determination of the pathological margins of laryngeal carcinoma

**DOI:** 10.1186/s12957-022-02788-8

**Published:** 2022-10-12

**Authors:** Jing Ke, Lifeng Jia, Yaqin Hu, Xu Jiang, Hailan Mo, Xiang An, Wei Yuan

**Affiliations:** 1 Chongqing Medical University, Chongqing, China; 2 Department of Otorhinolaryngology-Head and Neck Surgery, Chongqing Hospital, University of the Chinese Academy of Sciences (SCAS Chongqing), Chongqing, China; 3 Chongqing School, University of the Chinese Academy of Sciences (SCAS Chongqing), Chongqing, China; 4 Department of Otorhinolaryngology-Head and Neck Surgery, Chongqing General Hospital, Chongqing, China

**Keywords:** Laryngeal carcinoma, Pathological margins, Terahertz spectroscopy and imaging, Absorption coefficient, Refractive index

## Abstract

**Objective:**

Laryngeal cancer is a common malignancy in otorhinolaryngological head and neck surgery, accounting for approximately one-third of all head and neck malignancies. Terahertz time-domain spectroscopy (THz-TDS) has recently been found to be useful for the detection of tumors. This study was conducted to investigate the application of THz-TDS in the diagnosis of pathological resection margins of laryngeal cancer.

**Methods:**

Fresh laryngeal cancer tissues from 10 patients with laryngeal cancer were extracted, and after simultaneous HE staining and terahertz imaging, the tumor area, paracancerous area, and normal tissue area of each laryngeal cancer tissue sample were located under a microscope according to the pathological results of HE staining.

**Results:**

The shape contours of the tumor region revealed by terahertz imaging maps and HE staining were similar. In the terahertz spectrum in the frequency range of 0.5–1.9 THz, both the absorption coefficient and refractive index values followed the order tumor > para cancer > normal tissue, with statistically significant differences (*P* < 0.01). When the terahertz frequency was 1.5 THz, the absorption coefficient of terahertz light waves by laryngeal cancer tissue and the percentage of nuclei showed an extremely high positive correlation (*P* < 0.01, *r* = 0.971). In the frequency ranges of 0.5–1.2 THz and 1.6–1.9 THz, the absorption coefficients of the highly differentiated group were higher than those of the moderately differentiated group. In the frequency range of 1.2–1.6 THz, the results were reversed, with statistically significant differences (*P* < 0.05). In the frequency range of 0.5–1.9 THz, the highly differentiated group had a higher refractive index than the moderately differentiated group, with a statistically significant difference (*P* < 0.05).

**Conclusions:**

THz-TDS can be used to determine the pathological margins of laryngeal cancer based on the absorption coefficient and refractive index, and the magnitudes of the absorption coefficient and refractive index are related to the percentage of nuclei. The degree of differentiation of laryngeal cancer tissue can be assessed by THz-TDS. The study shows that the terahertz time-domain system is promising for applications in the diagnosis of laryngeal cancer, especially for the more accurate identification of intraoperative margins.

## Introduction

Laryngeal carcinoma is a common malignant tumor of the head and neck that accounts for approximately one-third of all malignant tumors of the head and neck. The 5-year survival rate for patients with laryngeal carcinoma is only 54.26–56.52% [1]. The onset of laryngeal carcinoma is obscure, and even distant metastasis occurs in the early stage [2]. Therefore, two-thirds of patients are in advanced stages (stages III and IV) when they go to the doctor for treatment [3]. Surgery is the main method for treating laryngeal carcinoma at present. Negative surgical margins are the main index for evaluating the complete resection of tumors, and they are an independent and the most important prognostic factor of head and neck malignant tumors [4]. It is important to decrease the recurrence rate and improve the long-term survival rate of patients with laryngeal carcinoma [5]; however, few methods are available that can help doctors judge surgical margins, and the use of frozen sections is almost the only method available for identifying negative surgical margins in head and neck surgery. It was found that [6] the recurrence rate of laryngeal carcinoma was 9–32% even when the surgical margins were frozen during the operation. This not only indicates that there are defects in the judgement of the resection margins of head and neck tumors intraoperatively using frozen sections but also suggests that it is of great clinical significance to find a new method for accurately evaluating the resection margins of head and neck tumors intraoperatively. Narrowband imaging, NIR autofluorescence, and terahertz techniques are innovative methods for intraoperative margin determination in recent years. However, the results of narrow-band imaging are closely related to the operator’s experience and technique, and there is a certain degree of error, while NIR autofluorescence needs further research for tumor diagnosis and is now more commonly used for thyroid and parathyroid studies [7, 8]. Therefore, terahertz technology is a better choice due to its unique optical advantages and the rapid development in oncology in recent years.

The terahertz technique is a detection technique with wide application prospects that emerged in the 1980s. In the past, research into terahertz was more focused on high-energy physical lasers, microwave radar, and data communications, with high scientific difficulty and huge investment costs. In recent years, with more efficient terahertz ray generators and detectors, researchers have begun to develop waveguides, filters, and beam splitters to manipulate terahertz waves for their use in biomedical applications. Terahertz waves are electromagnetic waves with frequencies in the range of 0.1–10 THz, between those of infrared and microwave waves [9]. Compared with traditional medical diagnostic methods, such as histopathology and imaging, the terahertz technique has the advantages of low energy, high resolution, broad spectral analysis ability, no need for marking, and no damage to the human body [10].

In the field of biomedicine, the application of terahertz technology has been widely studied. In particular, the terahertz technique has been used in many studies on tumors whose pathogenesis has not been fully understood. Studies at home and abroad on terahertz wave detection in skin cancer [11], breast cancer [12], brain glioma [13], colorectal cancer [14], gastric cancer [15], and other tumors reported that the response of tumor tissues to terahertz waves was different from that of normal tissues in a certain frequency band. At present, no study on the detection of laryngeal carcinoma by terahertz waves has been reported. Therefore, this study performed terahertz imaging and spectral detection of laryngeal carcinoma tissue and compared the results with pathological examination results. The aim of this study was to evaluate the real-time performance and accuracy of the THz-TDS for evaluating tumor resection margins.

## Materials and methods

### Sample collection and experimental procedures

Tissue samples from 10 patients who underwent laryngectomy in Chongqing People’s Hospital from May to September 2021 were collected for this 2021 study. The human samples involved in this study were ethically approved by Chongqing People's Hospital. The inclusion and exclusion criteria for the sample were as follows.

The inclusion criteria:

1. Patients who underwent open laryngeal cancer surgery, including total and partial laryngeal surgery.
2. Preoperative pathological biopsy results suggestive of squamous cell carcinoma.
3. Tumor tissue diameter ≥ 1 cm.

Exclusion criteria:

1. Patients with other tumors in combination.
2. Patients who are recurrent tumors.
3. Patients who have undergone radiotherapy and chemotherapy.

After HE staining and terahertz imaging, the areas of laryngeal carcinoma, adjacent tissue, and normal tissue were determined according to the pathological results of HE staining. Then, the terahertz transmission spectra of the three different types of tissue regions were analyzed.

### HE histopathology

**Fig. 1:**
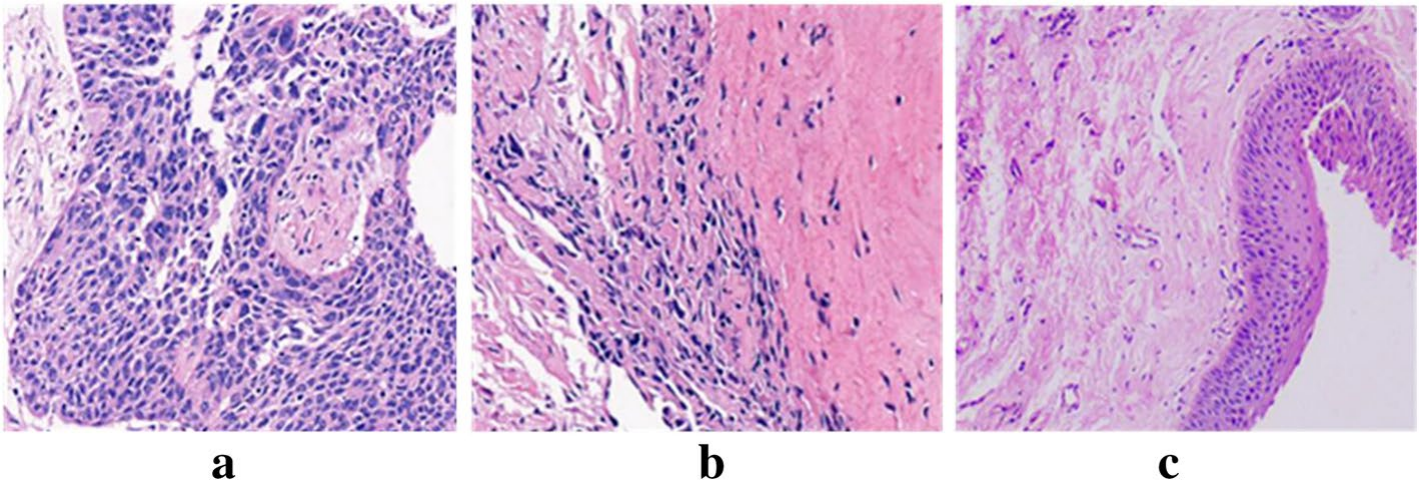
Histopathological analysis of human laryngeal carcinoma using HE-stained sections (× 200). **a** Laryngeal carcinoma: the tumor cell atypia is obvious, the arrangement is disorderly, the cell nuclei are large, and the pathological nuclear mitosis is increased. **b** Adjacent tissue: there is a clear dividing line between tumor tissue and normal tissue. **c** Normal tissue

Along the cross-section of each sample, 6-μm-thick tissue slices were taken and stained with HE. The results are shown in Fig. 1. Laryngeal carcinoma (a), adjacent tissue (b), and normal tissue (c) were found under the microscope.

### Transmission terahertz time-domain system detection

**Fig. 2:**
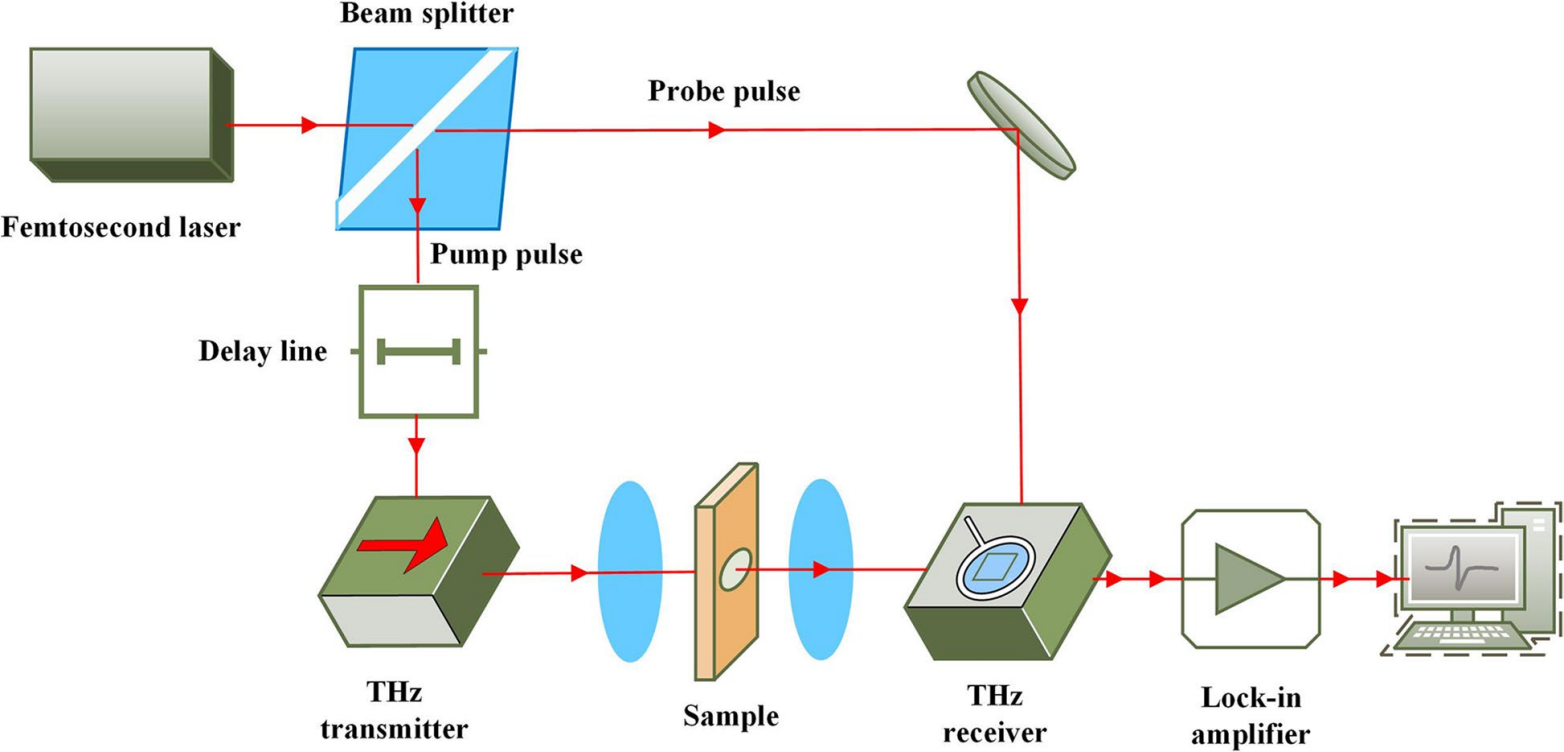
Experimental schematic diagram of the transmission terahertz time-domain spectroscopy system: The laser source emits a femtosecond laser pulse of a suitable wavelength. After passing through a spectroscope, it is divided into two beams: an emission beam and a detection beam. The emission beam generates a terahertz beam through a photoconductive antenna; it is then focused on the surface of the sample by a set of parabolic lenses, and the terahertz beam is collected by the detector through the sample under the control of an optical delay translation platform, focusing with a coherent detection beam on the detector for photoconductivity detection. Finally, the lock-in amplifier reads the sample information with a terahertz time-domain waveform

**Fig. 3:**
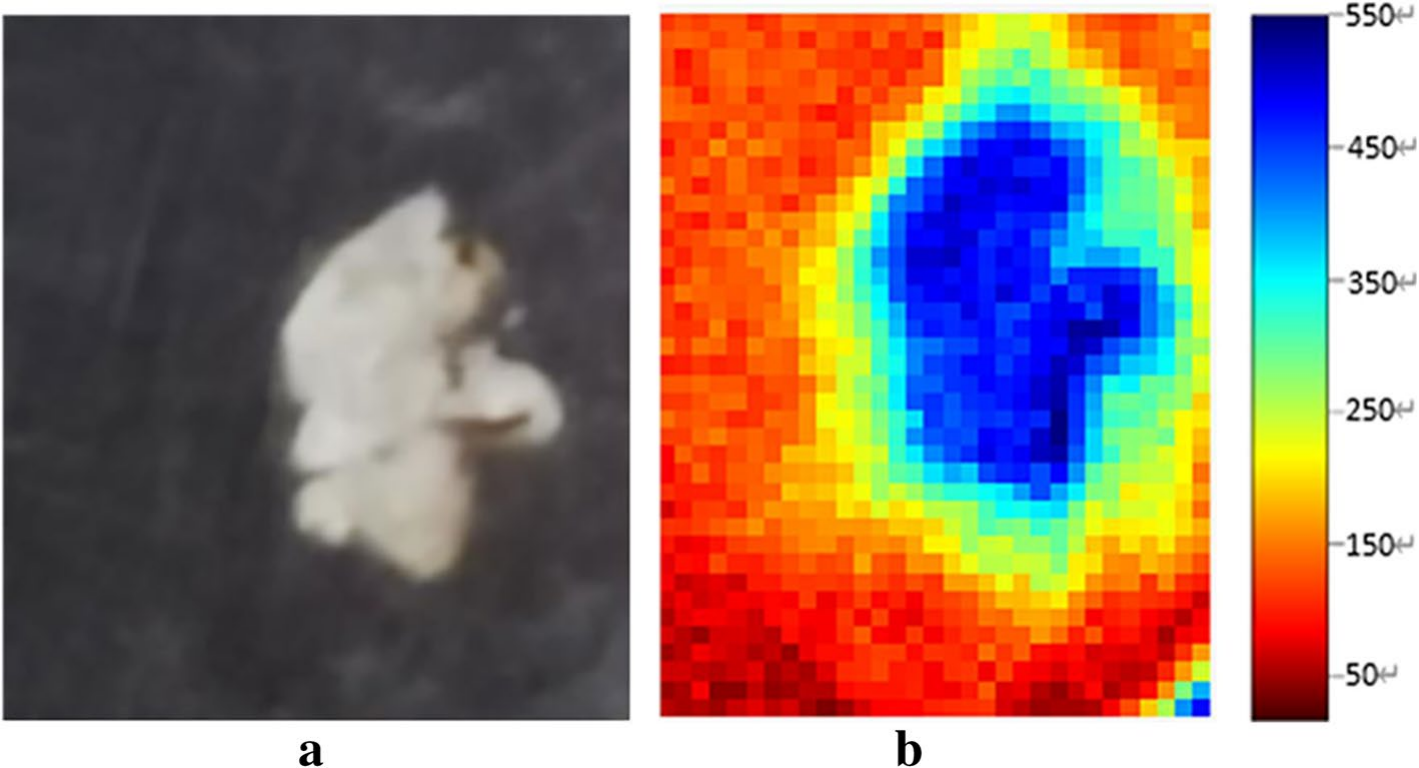
THz detection results of cancer tissue. **a** Optical image of a tissue section and **b** 1.5 THz frequency domain amplitude imaging map, in which blue and green areas represent tissue samples

At adjacent cross-sections, 100-μm-thick sections of the samples were sectioned for imaging detection of tumor and normal tissues using a terahertz time-domain system (Menlo Tera Sync, T-ray 5000). The optical geometry of this system is shown in Fig. 2. This system operates as follows: a laser source emits a femtosecond laser pulse of a suitable wavelength, which is divided into two beams after passing through a spectroscope: an emission beam and a detection beam. A terahertz transmitted beam is generated by a photoconductive antenna, which is then focused on the surface of the sample by a set of parabolic mirrors. The terahertz beam passing through the sample is collected by a detector under the control of an optical delay translation platform, focusing with a coherent detection beam on the detector for photoconductivity detection. Finally, the lock-in amplifier reads the sample information with the terahertz time-domain waveform. The experimental steps were as follows: (a) the THz test mode was set to the transmission mode. (b) The prepared tissue sample was placed on the sample carrier. The basic terahertz signal intensity at the sample decreased, the center position of the sample was estimated, and a rectangular area of 1 cm × 1 cm was formed. A 1 mm moving step was used for coarse scanning. (c) Real-time reconstruction of the peak value of the terahertz spectrum from the rough scan image was performed to find the specific location of the sample, and then a 0.25 mm moving step was used to carry out fine scanning. (d) The terahertz electric field signal was collected using LabVIEW control software, and MATLAB 2021 was used to carry out spectrum analysis and imaging. An optical image of our tumor tissue section sample is shown in Fig. 3a, and the radiometric imaging map in the 1.5 THz frequency domain in Fig. 3b reflects the terahertz wave absorption properties of the sample in the 1.5 THz frequency band. Furthermore, we compared these results to the results of HE staining to extract the terahertz time-domain signals of laryngeal carcinoma tissue and normal tissue for analysis: from the two regions, 10 THz signals were extracted, averaged, and converted into the corresponding frequency domain data by Fourier transform.

### Data analysis

Difference analysis of the absorption coefficient and refractive index values detected by terahertz transmission spectroscopy was conducted among three regions, namely, the tumor, paracancerous, and normal tissue regions. The formula for calculating the absorption coefficient α (ω) and the refractive index *n* (ω) of a sample is as follows:

1 $$n\left(\omega \right)=\frac{c\varphi \left(\omega \right)}{\omega d}+1$$

2 $$k\left(\omega \right)=\ln \left[\frac{4n\left(\omega \right)}{A\left(\omega \right){\left(n\left(\omega \right)+1\right)}^2}\right]\frac{c}{\omega d}$$

3 $$\upalpha \left(\omega \right)=\frac{2k\left(\omega \right)\omega }{c}$$

where ω is the angular frequency, *C* is the vacuum speed of light, *D* is the thickness of the sample, a (ω) is the module of the sample signal and the reference signal, and φ (ω) represents the phase of the ratio of the sampled signal to the reference signal [16].

The correlation between the percentage of nuclei in the tumor area and its terahertz absorption coefficient was analyzed. It has been shown that terahertz spectroscopy can be used to detect the differences between nucleic acids [17] and proteins [18, 19]. Since most of the nucleic acids are located in the nucleus, the relationship between the percentage of nuclei and the absorption coefficient of the terahertz transmission spectrum was analyzed. The percentage of nuclei was calculated using the software Image-Pro Plus 6.0. The relationship between the absorption coefficient and nuclear area was analyzed using IBM SPSS Statistics 21.

The differences in the terahertz absorption coefficient values of tumor tissues with different differentiation degrees were analyzed. We studied that the higher the degree of differentiation was, the lower the malignancy degree of the tumor, the more mature the tumor cells, the more viscous the cytoplasm, and the higher the water content of the cells. Only moderately and highly differentiated tumor tissues were collected in this study, so this experiment only compared the differences between the two.

The data were analyzed using Origin 2021 statistical software. The data were filtered using MATLAB 2021. Principal component analysis (PCA) was used to evaluate the spectral differences among the tumor, adjacent tissue, and normal tissue.

## Results

### Comparison of contour shapes of tumor areas

**Fig. 4:**
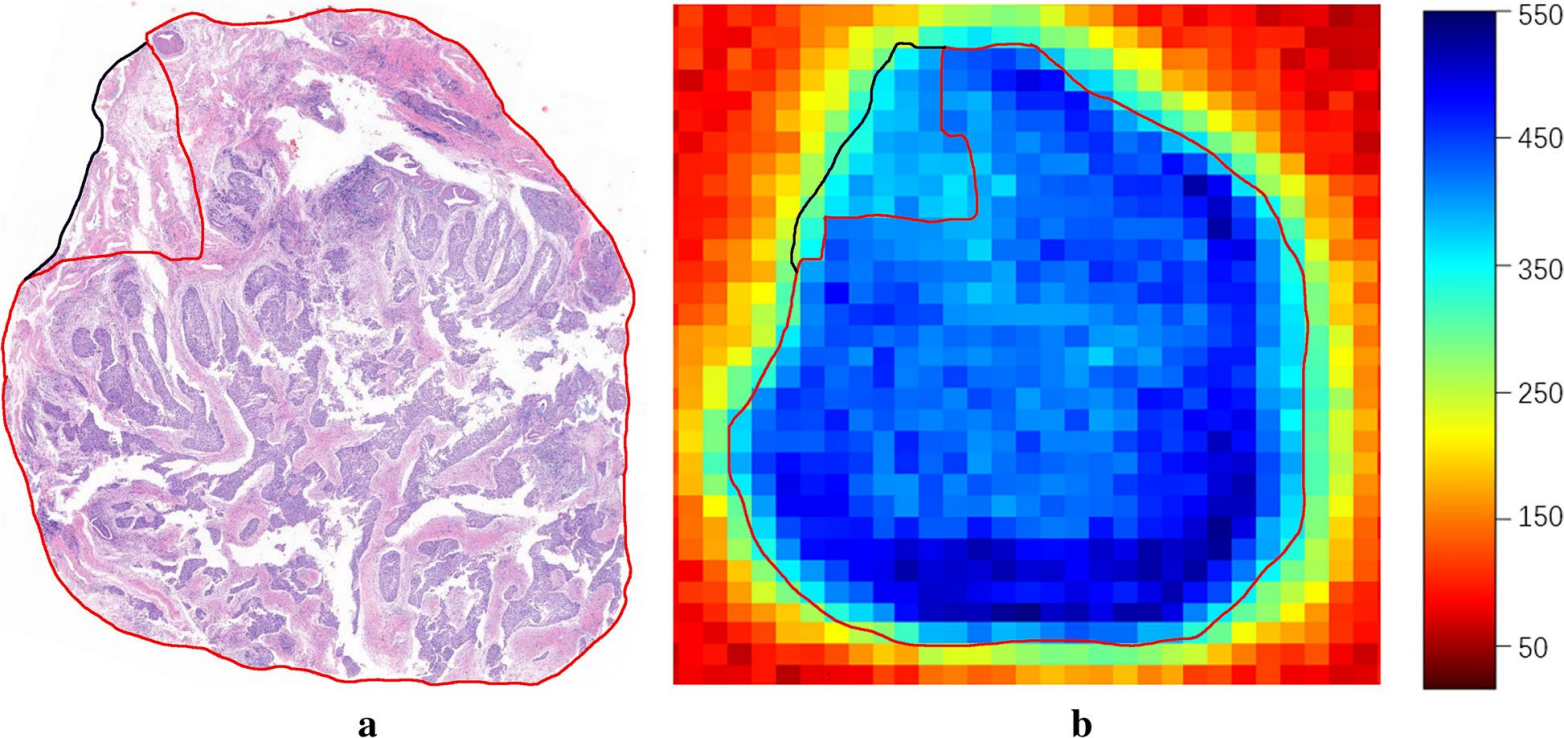
Tumor contour comparison between histopathologic and terahertz images. **a** HE-stained pathology map of laryngeal carcinoma tissue and **b** terahertz imaging map of the amplitude in the 1.5 THz frequency domain. In **a**, the red-lined area represents the tumor tissue and the black-lined area represents the paracancerous tissue. In **b**, the darker the color, the higher the absorption value; the dark blue area represents the tumor area, the light blue represents the paracancerous tissue and the green represents normal tissue

The contour shapes of tumor tissue regions obtained by terahertz transmission imaging and from pathological images were compared. In our study, we used the THz technique to perform terahertz imaging of laryngeal carcinoma tissue samples and compared the results with the histopathological findings. Figure 4a shows the results of HE staining. Figure 4b shows the results of imaging at a frequency of 1.5 THz, with blue representing the region of tumor cells. The dotted line in the image shows the tumor cell area. The shapes and contours of the two are roughly the same.

### Analysis of the differences in the absorption coefficient and refractive index

**Fig. 5:**
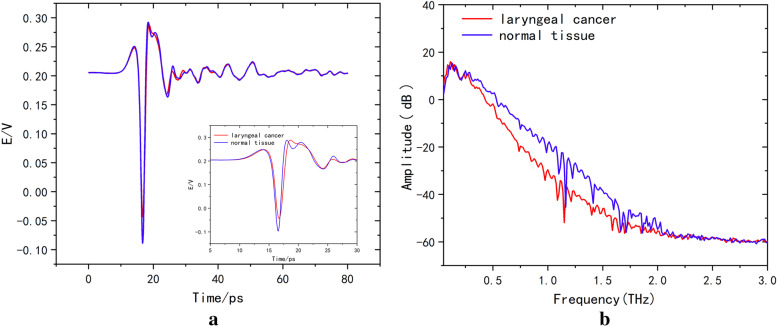
The THz spectrum of laryngeal carcinoma tissue is different from that of normal tissue (red represents laryngeal carcinoma tissue, and blue represents normal tissue). **a** Time domain: the time-domain spectra of laryngeal carcinoma tissues were different from those of normal tissues. The time of the wave peak in laryngeal carcinoma tissues was relatively delayed compared with that in normal tissues, and the peak value (wave peak-wave trough) from laryngeal carcinoma tissues to normal tissues increased from 0.33 to 0.38. **b** Frequency domain: In the frequency range of 0.25–2.5 THz, the transmission of THz waves in laryngeal carcinoma tissue was lower than that in normal tissue

**Fig. 6:**
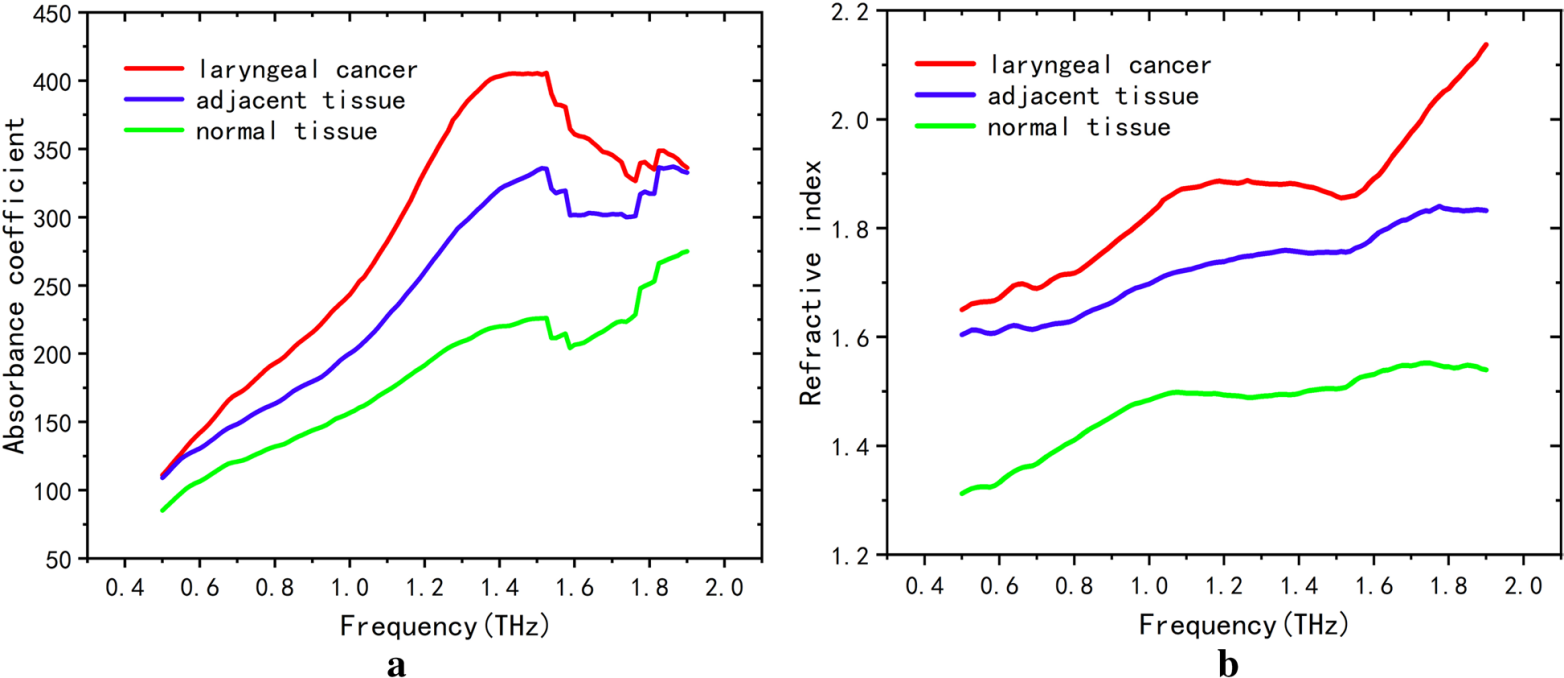
Comparison of terahertz optical parameters of laryngeal carcinoma, para-cancer and normal tissues (red represents laryngeal carcinoma, blue represents para-cancer, and green represents normal tissues) revealed significant differences among them: laryngeal carcinoma > adjacent tissue > normal tissue. **a** Absorption coefficient and **b** refractive index

**Fig. 7:**
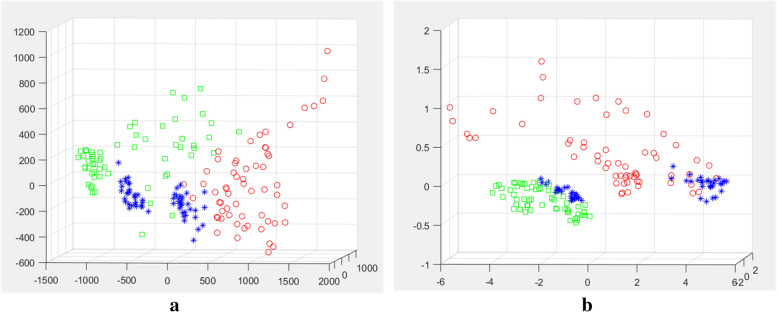
PCA score plots of terahertz optical parameters of laryngeal carcinoma, adjacent tissue, and normal tissue (red represents laryngeal carcinoma tissue, blue represents paracancerous tissue, and green represents normal tissue; three principal components were extracted, PC1 + PC2 + PC3 = 0.90). Laryngeal tumor, adjacent tissue, and normal tissue absorption coefficient values are distributed in different areas, which shows that the three groups can be well distinguished by the absorption coefficient. For the refractive index, the boundary between normal tissue and tumor tissue is obvious, but they are still distributed in different areas, which shows that the refractive index can be used to well distinguish between the three groups. **a** Absorption coefficient and **b** refractive index

**Fig. 8:**
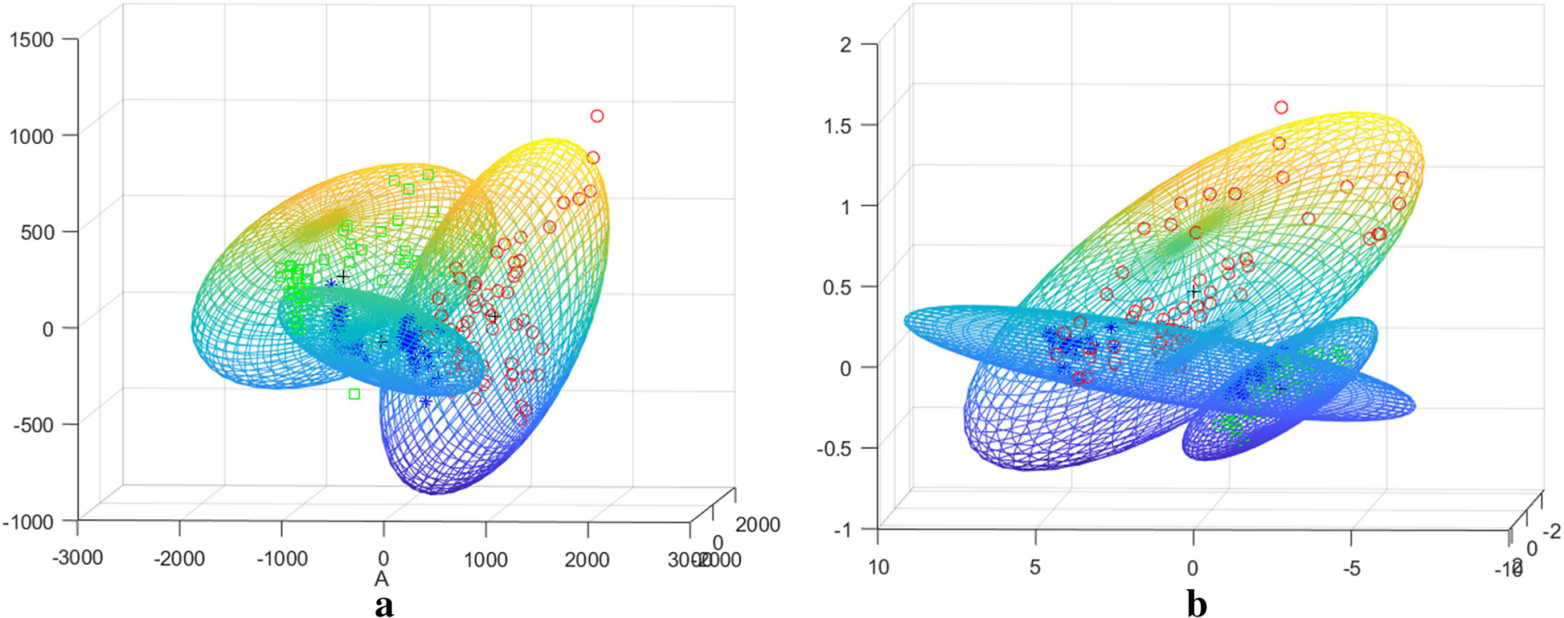
PCA 95% confidence ellipses of terahertz optical parameters for laryngeal carcinoma tissue, paracancerous tissue, and normal tissue (red represents laryngeal carcinoma tissue, blue represents paracancerous tissue, and green represents normal tissue). **a** Absorption coefficient and **b** refractive index

Terahertz time domain signals from extracted laryngeal cancer and normal tissue were analyzed as shown in Fig. 5a, and the time-domain spectra of laryngeal carcinoma tissues were different from those of normal tissues. The wave peaks in the laryngeal carcinoma tissues were relatively delayed compared to those in the normal tissues. The peak value (peak-trough) from laryngeal carcinoma to normal tissue increased from 0.33 to 0.38. As shown in Fig. 5b, in the frequency range of 0.25–2.5 THz, the transmission of terahertz waves from laryngeal carcinoma tissues was lower than that from normal tissues. The difference in the absorption coefficient values in the range of the effective spectrum was statistically significant (*p* < 0.001). Difference analysis of the absorption coefficient and refractive index detected by terahertz transmission spectroscopy among the tumor, adjacent tissue, and normal tissue regions was conducted. We Fourier transformed the spectra detected by THz-TDS to the corresponding frequency spectrum data, and the absorption coefficient and refractive index of the specimen were calculated using the specified formulas. The results are shown in Fig. 6. The red curve is the terahertz spectrum of the tumor tissue, the blue curve is the terahertz spectrum of the paracancerous tissue, and the green curve is the terahertz spectrum of the normal tissue. According to the terahertz absorption coefficient spectrum (a), the absorption coefficients followed the ordering tumor > para-cancer > normal tissue. With increasing detection frequency, the absorptions of the three compounds increased and peaked at 1.5 THz. The terahertz absorption coefficients of the tumor, paracancerous, and normal tissues were significantly different (*p* < 0.01). Similar to the absorption coefficient, the refractive indices in the refractive index spectrum (b) followed the ordering tumor > paracancerous tissue > normal tissue and differed significantly (*p* < 0.01) among the regions. The absorption coefficient and refractive index values of the tumor, adjacent tissue, and normal tissue were analyzed by principal component analysis (PCA) in patients with laryngeal carcinoma, as shown in Fig. 7, in which red indicates tumor, blue indicates adjacent tissue, and green indicates normal tissue. As shown in Fig. 8, the tumor, adjacent tissue, and normal tissue were distributed in different absorption coefficient value regions, indicating that the three could be well distinguished by the absorption coefficient; however, for the refractive index, the normal tissue and the tumor were well separated, and the para-cancerous tissue was close to them, but it was still distributed in different areas.

### Correlation analysis between the percentage of nuclei and absorption coefficient

**Fig. 9:**
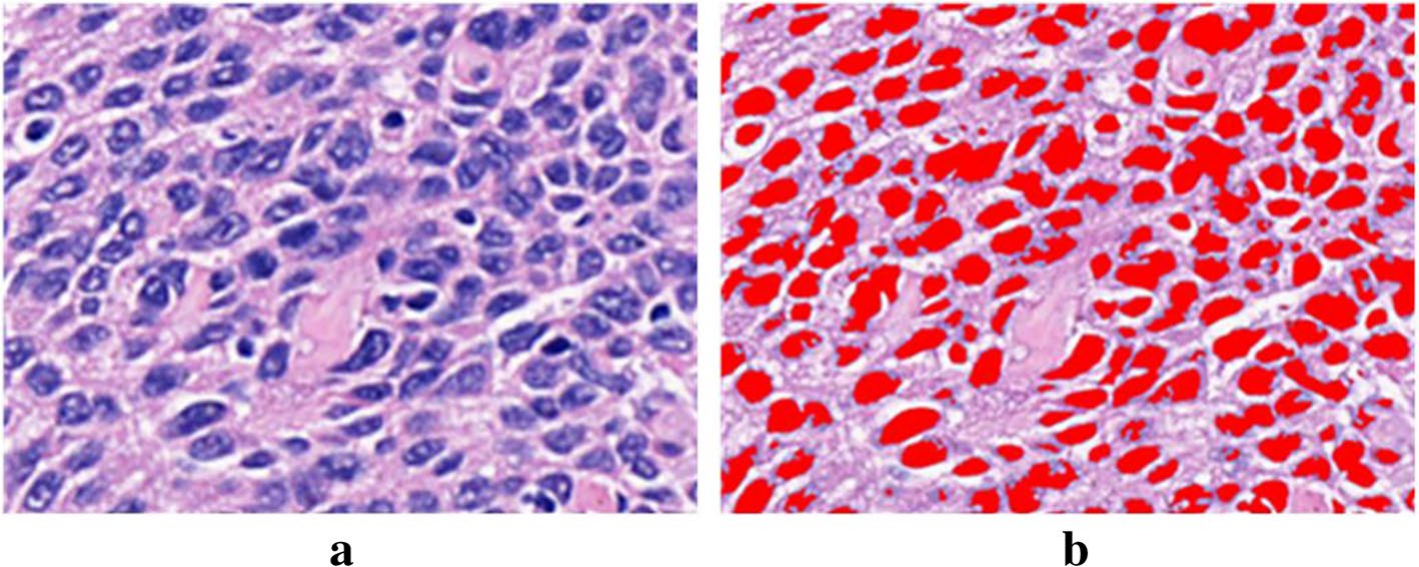
Tumor pathology original image and image after processing with Image-Pro Plus. **a** Laryngeal carcinoma pathology image (the blue–purple color represents the nucleus, × 400) and **b** Image-Pro Plus processed image (the red colour represents the nucleus, × 400)

**Fig. 10:**
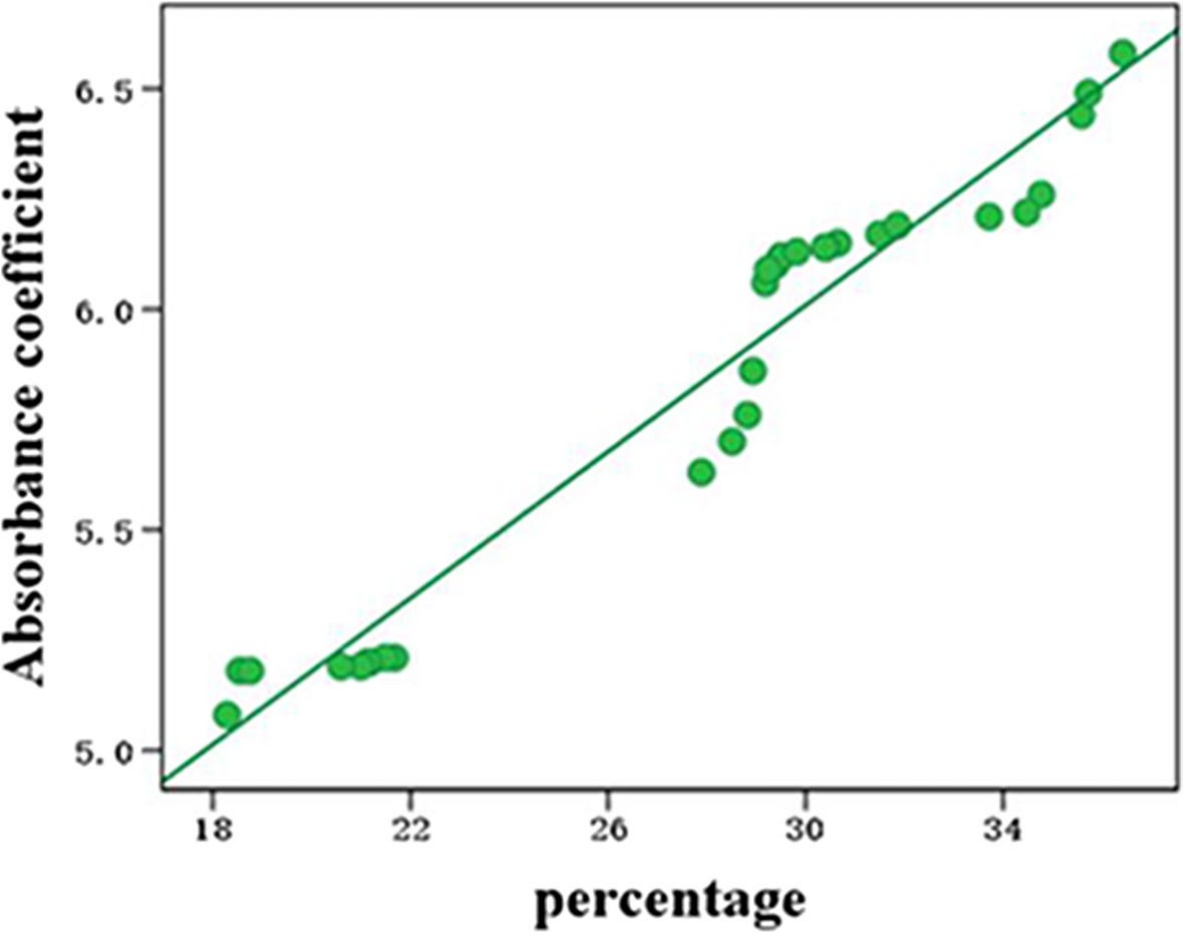
Linear relationship between the THz absorption coefficient and nuclear percentage of tumor tissue at 1.5 THz: there was a high positive correlation between the absorption coefficient and the nuclear area ratio (*p* < 0.01, *r* = 0.971)

The correlation between the percentage of nuclei in tumor tissue and the absorption coefficient of the THz spectrum was analyzed. The correlation between the absorption coefficient of the terahertz spectrum and the percentage of nuclei was studied when the frequency was 1.5 THz. Tumor pathology maps were processed using Image-Pro Plus 6.0, and the results are shown in Fig. 9. A correlation analysis was performed with IBM SPSS Statistics 21 software, and the relationship is shown in Fig. 10, with a very high positive correlation between the absorption coefficient and the nuclear area ratio (*p* < 0.01, *r* = 0.971).

### Differential analysis of moderately and highly differentiated tumors

**Fig. 11:**
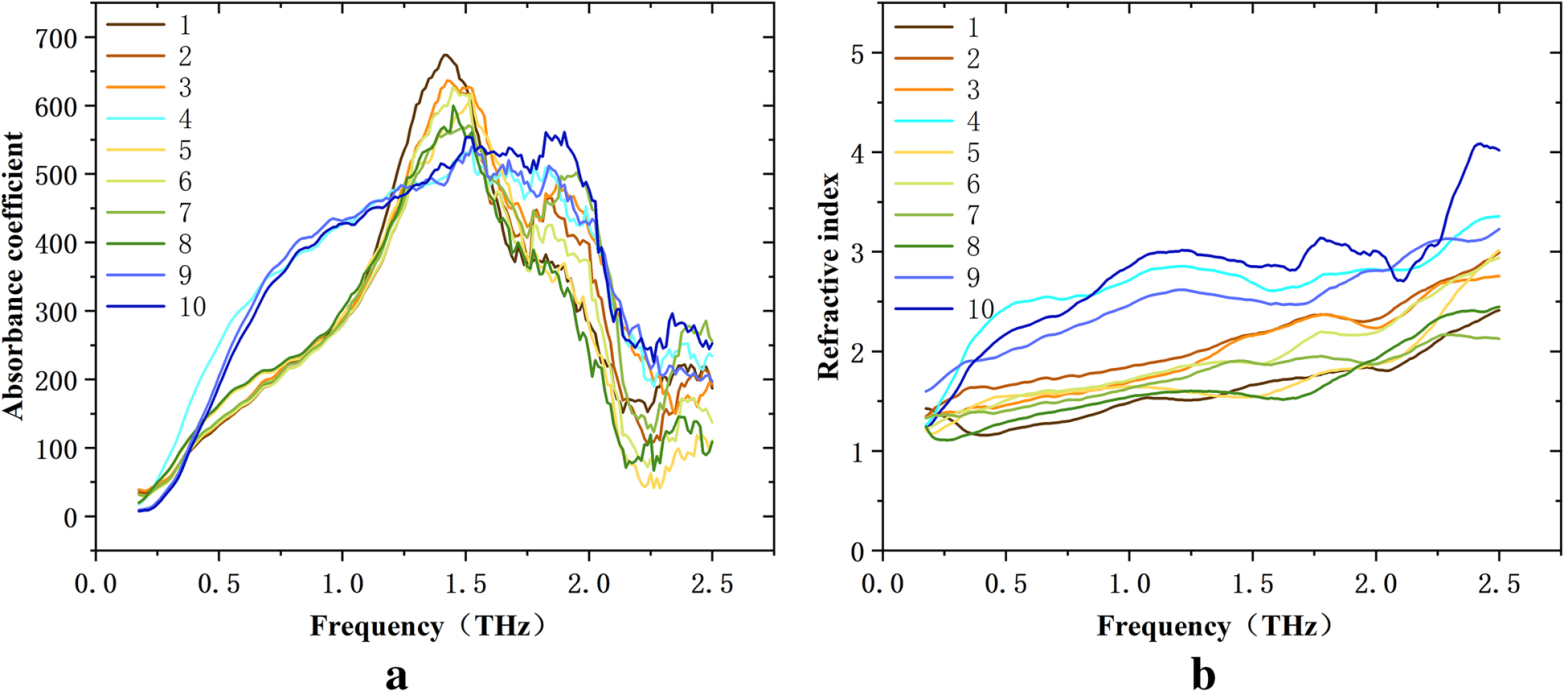
Comparison of terahertz optical parameters between moderately differentiated and highly differentiated laryngeal carcinoma tissues: there were significant differences in the THz absorption coefficient and refractive index (moderately differentiated: 1-3, 5-8; highly differentiated: 4, 9, 10; *p* < 0.05). **a** Absorption coefficient and **b** refractive index

**Fig. 12:**
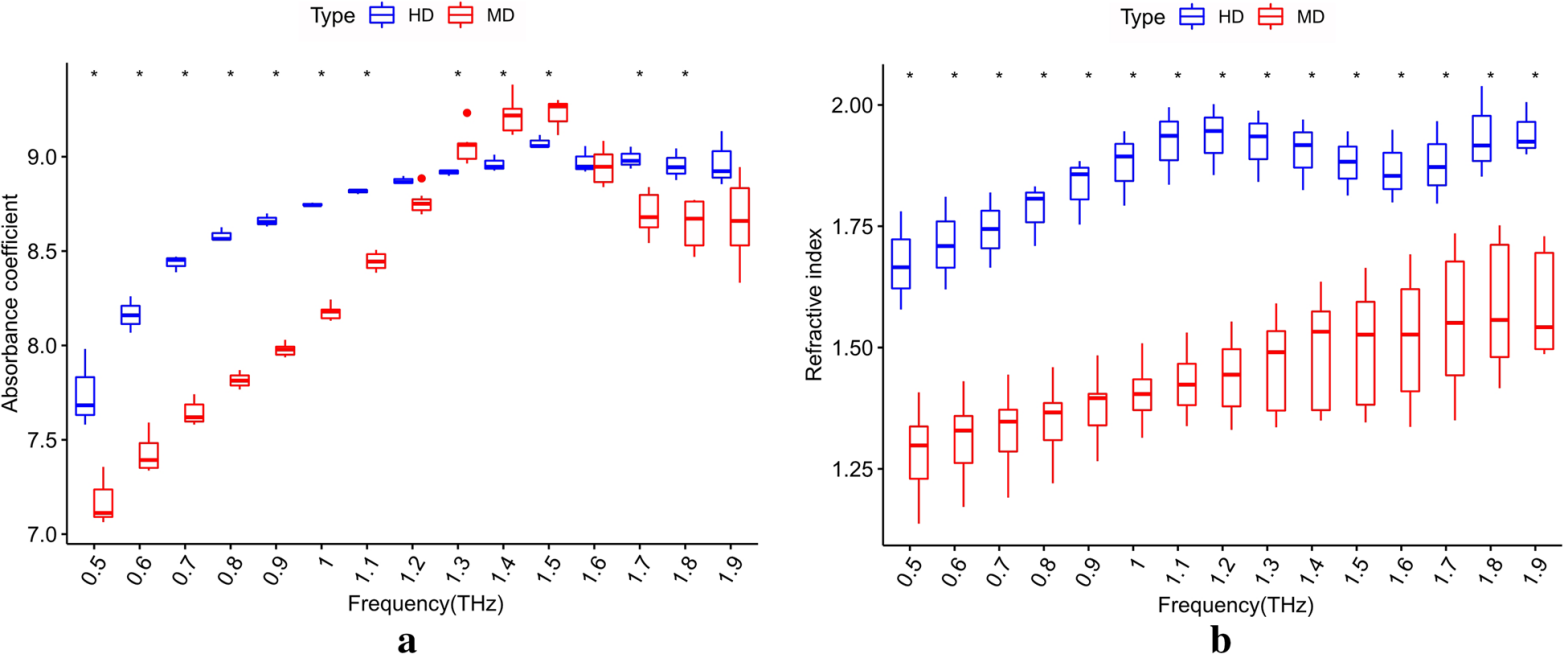
THz optical parameter error analysis of moderately differentiated and highly differentiated laryngeal carcinoma tissues. HD: highly differentiated; MD: moderately differentiated; *p < 0.05. **a** Absorption coefficient and **b** refractive index

An analysis of the differences in terahertz optical parameters between moderately differentiated and highly differentiated laryngeal carcinoma tissues was conducted. There were 7 cases in the moderately differentiated group and 3 cases in the highly differentiated group. The average absorption coefficient and refractive index values of the two groups at different frequencies were compared, as shown in Fig. 11. The absorption coefficient and refractive index in the hertz frequency range were significantly different between the two groups (*p* < 0.05). The results were analyzed in terms of the error, as shown in Fig. 12. In the frequency ranges of 0.5–1.2 THz and 1.6–1.9 THz, the absorption coefficient of the highly differentiated group was higher than that of the moderately differentiated group, and in the frequency range of 1.2–1.6 THz, the results were reversed. In the frequency range of 0.5–1.9 THz, the refractive index of the highly differentiated group was higher than that of the moderately differentiated group (*p* < 0.05).

## Discussion

In this study, terahertz transmission spectroscopy was used to detect fresh laryngeal carcinoma tissue. In our pre-experiment, we randomly selected 3 groups of laryngeal carcinoma tissue samples and examined them with THz spectroscopy and imaging at thicknesses of 80, 100, 150, and 200 μm. The absorption coefficient and refractive index values of the three tissue regions were calculated by terahertz transmission spectrum detection, and for imaging at a thickness of 100 μm, the experimental error was the smallest. Therefore, in the follow-up experimental study, specimens of 100 μm thickness were used for detection.

The imaging results for the same specimen differed among frequencies. Theoretically, the spatial resolution of terahertz waves is limited by the diffraction limit, so the higher the frequency is, the higher the spatial resolution, and the better the imaging effect should be. However, this is not the case because as the frequency increases, the absorption coefficient of the sample also increases, which leads to decreases in the THz wave penetration depth and signal-to-noise ratio [20], which affect the clarity of the THz image. Therefore, we need to select a suitable frequency to clearly show the outline of the tumor area. By screening the imaging images of all samples at various frequencies, it was found that from the images of all samples at 1.5 THz, we could clearly identify the outline of the tumor area; thus, the images with a frequency of 1.5 THz were compared with the pathological images. In fresh laryngeal carcinoma tissue, terahertz imaging was used to identify the tumor contour and size, which were largely similar to the pathological results of HE staining. This shows that terahertz imaging detection has very high accuracy.

The terahertz waves in this experiment were in the range of 0.175–4.5 THz. We found that the terahertz spectra in the frequency range of 0.5–1.9 THz were significantly different among the three tissue regions. The refractive index and the absorption coefficient increased with increasing frequency, and the refractive index and absorption coefficient of the tumor for terahertz waves were larger than those of normal tissue and adjacent tissue. We attributed this to the following: because metabolism is vigorous in tumor tissue areas, the amounts of protein and nucleic acid macromolecules increase relatively, and lipids provide the material and the energy foundation for the formation of massive molecules, terahertz waves are sensitive to the structural changes of biomacromolecules in tissues; in addition, the content of organelles in tumor regions is increased because of the unlimited proliferation of tumor cells, which leads to a corresponding increase in moisture, which is detected by terahertz waves [21]. This also explains why, in this experiment, when we randomly selected 30 tumor regions of 10 tumor samples for analysis, the results showed that the higher the proportion of nuclei was, the higher the absorption coefficient of terahertz waves in laryngeal carcinoma tissue. The less differentiated tumor cells are, the more malignant they are. The matrix and nucleus become thin due to immature growth, leading to a decrease in water content, which results in significant differences in the absorption coefficient and refractive index of terahertz waves in tumor cells with different levels of differentiation. The study showed that the refractive index of the moderately differentiated group was always lower than that of the highly differentiated group, but the THz wave absorption coefficient of the moderately differentiated group was always higher than that of the highly differentiated group in the range of 1.2–1.6 THz. In this range of frequencies, the matrix and nucleus of the intermediate group were thin, causing the terahertz waves to pass faster, creating a superposition effect between the waves and making the absorption value of the intermediate group higher, but outside this range, the absorption coefficient of the highly differentiated group was always higher than that of the moderately differentiated group.

THz-TDS is accurate in the diagnosis of cancer and compares favorably with conventional CT, histopathology, endoscopy, etc. [22]. The terahertz technique takes less time (an average specimen takes approximately a minute and a half), and the sample processing is simple (just obtaining a slice of fresh tissue). The diagnosis is more intuitive and based on data, and less subjective judgement is involved. Compared with liver cancer, bone cancer, lung cancer, brain tumors, and kidney cancer, among other cancers, laryngeal carcinoma is relatively superficial and located in an open space, in direct proximity to the tumor tissue detection and treatment; hence, the skin, muscle, fat, and other tissues cause less interference. Our study confirmed that the application of the THz technique in the detection of laryngeal carcinoma is feasible, which provides a theoretical basis for its application in laryngeal carcinoma. It is important to note that our study has limitations and is a small sample, and further expansion of the sample size is needed in subsequent studies.

## Conclusions

Terahertz imaging has been applied to the clinical research of breast cancer abroad [23]. This technique, combined with the use of endoscopic devices for in vivo boundary coverage, may help physicians in future radiation oncology disciplines perform more accurate target delineation and reduce damage to other normal tissues, and it may facilitate the judgement of surgical margins [24]. Research into terahertz endoscopy systems has made some progress [14]. The four steps required for successful terahertz endoscopic imaging are to obtain the ideal imaging frequency for the system, to evaluate the underlying contrast, to test a flexible terahertz waveguide for use as an endoscope, and to demonstrate terahertz waveguide-based tissue imaging. Although the reports available are all about the digestive tract, we believe that an ENT endoscopic system is based on the same principles as an endoscopic system for the digestive tract, and therefore terahertz endoscopic imaging in the pharynx is achievable. However, the next difficulty to be faced in realizing this technology is the flexible terahertz waveguide that can be used in the endoscopic system, which is subject to further development of terahertz technology. In the future, we can combine the terahertz technique with an endoscopic system for the diagnosis and treatment of nasopharyngeal carcinoma and pharynx carcinoma and improve the rate of complete tumor resection, prolonging the life of the patient. Therefore, the application of terahertz imaging in cancer detection is both an opportunity and a challenge [21]. At present, research in this area is still in its infancy.

## Data Availability

The datasets supporting the conclusions of this article are included within the article. The first author can be contacted to provide raw data if required.

## References

[1] Li MM, Zhao S, Eskander A, Rygalski C, Brock G, Parikh AS (2021). Stage migration and survival trends in laryngeal cancer. Ann Surg Oncol.

[2] Koontongkaew S (2013). The tumor microenvironment contribution to development, growth, invasion and metastasis of head and neck squamous cell carcinomas. J Cancer.

[3] Steuer CE, El-Deiry M, Parks JR, Higgins KA, Saba NF (2017). An update on larynx cancer. CA Cancer J Clin.

[4] Tassone P, Savard C, Topf MC, Keane W, Luginbuhl A, Curry J (2018). Association of positive initial margins with survival among patients with squamous cell carcinoma treated with total laryngectomy. JAMA Otolaryngol Head Neck Surg.

[5] Dinc ASK, Cayonu M, Boynuegri S, Sahin MM, Eryilmaz A (2019). The effect of surgical margin positivity on survival in laryngeal cancer surgery. Ann Ital Chir.

[6] Beibei Y, Rong Y, Yunfei Y, Wenchao Z (2021). Research progress regarding surgical margins, molecular margins, and prognosis of laryngeal carcinoma. Ear Nose Throat J.

[7] Di Marco AN, Palazzo FF (2020). Near-infrared autofluorescence in thyroid and parathyroid surgery. Gland Surg.

[8] Squires MH, Jarvis R, Shirley LA, Phay JE (2019). Intraoperative parathyroid autofluorescence detection in patients with primary hyperparathyroidism. Ann Surg Oncol.

[9] Fedorov VY, Tzortzakis S (2020). Powerful terahertz waves from long-wavelength infrared laser filaments. Light Sci Appl.

[10] Sun L, Zhao L, Peng RY (2021). Research progress in the effects of terahertz waves on biomacromolecules. Mil Med Res.

[11] Amini T, Jahangiri F, Ameri Z, Hemmatian MA (2021). A review of feasible applications of THz waves in medical diagnostics and treatments. J Lasers Med Sci.

[12] Wang L. Terahertz imaging for breast cancer detection. Sensors (Basel, Switzerland). 2021;21(19):6465. 10.3390/s21196465 PMC8512288 34640784

[13] Wu L, Xu D, Wang Y, Liao B, Jiang Z, Zhao L (2019). Study of in vivo brain glioma in a mouse model using continuous-wave terahertz reflection imaging. Biomed Opt Express.

[14] Doradla P, Joseph C, Giles RH (2017). Terahertz endoscopic imaging for colorectal cancer detection: current status and future perspectives. World J Gastrointest Endosc.

[15] Grigorev R, Kuzikova A, Demchenko P, Senyuk A, Svechkova A, Khamid A, et al. Investigation of fresh gastric normal and cancer tissues using terahertz time-domain spectroscopy. Materials (Basel, Switzerland). 2019;13(1):85. 10.3390/ma13010085 PMC6981444 31877967

[16] Zhang J, Liu Z, Zhang P, Bai Z (2019). Terahertz-based spectroscopy and imaging for prostate cancer detection. Chin J Med Phys.

[17] Tang M, Zhang M, Yan S, Xia L, Yang Z, Du C (2018). Detection of DNA oligonucleotides with base mutations by terahertz spectroscopy and microstructures. Plos one.

[18] Yang Z, Tang D, Hu J, Tang M, Zhang M, Cui HL (2021). Near-field nanoscopic terahertz imaging of single proteins. Small (Weinheim an der Bergstrasse, Germany).

[19] Han X, Yan S, Zang Z, Wei D, Cui HL, Du C (2018). Label-free protein detection using terahertz time-domain spectroscopy. Biomed Opt Express.

[20] Li D, Yang Z, Fu A, Chen T, Chen L, Tang M (2020). Detecting melanoma with a terahertz spectroscopy imaging technique. Spectrochim Acta A Mol Biomol Spectrosc.

[21] Yu L, Hao L, Meiqiong T, Jiaoqi H, Wei L, Jinying D (2019). The medical application of terahertz technology in non-invasive detection of cells and tissues: opportunities and challenges. RSC Adv.

[22] Yu C, Fan S, Sun Y, Pickwell-Macpherson E (2012). The potential of terahertz imaging for cancer diagnosis: a review of investigations to date. Quant Imaging Med Surg.

[23] Vohra N, Bowman T, Bailey K, El-Shenawee M. Terahertz imaging and characterization protocol for freshly excised breast cancer tumors. J Vis Exp. 2020;158:e61007. 10.3791/61007 PMC7179081 32310233

[24] Joseph CS, Yaroslavsky AN, Neel VA, Goyette TM, Giles RH (2011). Continuous wave terahertz transmission imaging of nonmelanoma skin cancers. Lasers SurgMed.

